# The Impact of the Doctor-Patient Relationship on the Treatment Goal in Rheumatology

**DOI:** 10.31138/mjr.110823.dpr

**Published:** 2024-02-01

**Authors:** Panagiota Tsatsani, Aspasia Goula, Maria-Aggeliki Stamouli, Sotiris Soulis

**Affiliations:** 1 KAT General Hospital of Athens, Athens, Greece,; 2 Department of Business Administration, University of West Attica, Athens, Greece

**Keywords:** rheumatic diseases, doctor-patient relationship, quality of life, duration of relationship with the doctor

## Abstract

**Objectives:**

To assess the perceptions of patients with rheumatic diseases about the doctor-patient relationship and the impact on their treatment and their quality of life.

**Methods:**

A quantitative study collecting data from patients with rheumatic diseases using the following tools: (a) the Doctor-Patient Relationship Assessment Questionnaire-16 (DoPRAQ-16), assessed the quality of doctor-patient relationship, (b) the Short Form 36 (SF-36) assessed the quality of life of patients, and (c) the Health Assessment Questionnaire (HAQ), assessed the functional ability of patients. From the statistical analysis, it appears that there is no linear correlation between the DoPRAQ-16 scales and the dimensions of the SF-36 Health Survey, except for the scale of negative emotions and the dimension of Physical Functioning. The nonparametric Kruskal-Wallis H test was performed to investigate the existence of statistically significant differences between the categories of duration of the relationship with the doctor to Physical Functioning, Physical Role, Emotional Role, and Social Functioning. The test was significant (p<0.05) for the dimensions of Body Role and Social Functioning.

**Conclusions:**

Patients with long term relationship with the doctor have better health quality in the dimension of Physical Role and Social Functioning compared to people whose relationship with the doctor lasts fewer years.

## INTRODUCTION

Rheumatic diseases are among the most common chronic diseases. The onset of a chronic disease is often considered to be a situation that brings severe changes in a patient’s lifestyle with unexpected consequences such as limited physical activity. These inflammatory diseases are not directly life-threatening but affect patients’ physical, mental, and social function resulting in low health-related quality of life (QoL).^1,2^ Thus, nowadays the main goal of a therapeutic procedure should be the improvement of health-related quality of life.

The health-related quality of life (HRQL) is an important index of the burden of rheumatic disease. Patients suffering from musculoskeletal diseases cannot bathe or dress themselves. Furthermore, other simple tasks such as walking can be difficult or impossible for them. Pain, fatigue, and physical limitations affect psychosocial functioning and patients develop long-term anxiety and depression.^3,4^ Health professionals should be particularly sensitive to the mental stress of the patient with chronic rheumatic diseases.

The role of the rheumatologist in helping the patient to accept the diagnosis, to comply with medical instructions, to manage negative emotions and to set goals for his future is particularly important. The therapist can help patients to manage the chronic disease through a proper communication development. Szasz and Hollender^5^ suggested that the most appropriate type of doctor-patient relationship is the “mutual participation” model, which means that both parties share responsibility for planning and implementing a treatment.

It is known that a balanced doctor-patient relationship offers better treatment results. Patients’ expectations reveal their need for human approach and a more personalised treatment in every medical visit. The modern medical practice focuses not only on improving the life expectancy of the patient and reducing their symptoms, but also on their wishes and needs.

From 1912, Freud^6,7^ spoke of the “therapeutic alliance of patient and doctor”. According to this approach, patient and doctor agree that they should ally themselves in order to achieve better therapeutic results in the face of the patient’s problems.

Nowadays, as human relationships become difficult, the doctor-patient relationship needs more attention and effort from both sides.^8^ Proper communication, mutual respect and solidarity must characterise this specific relationship.

Patients are no more passive receivers of medical care, but they take active part in the process. In order to proceed to treatment, absolute priority must be given to the development of trust and cooperation with the therapist. The doctor must create the appropriate conditions in which the patients are integrated and take part in the procedure of recognising the problems, plan the necessary therapy and estimate the outcomes of care.^9,10^

The effective communication determines the degree of patient compliance to medical instructions. High levels of trust to the rheumatologist combined with active patient participation in the decision-making process leads to low disease activity, less side effects, and greater satisfaction from therapy. Furthermore, help patients to manage their disease easily and be more optimistic about the future. Successful treatment of chronic rheumatic diseases requires a combination of medical therapy and psychological support. The emotional relationship between doctor and patient plays a significant role in order to achieve this goal. There is no doubt that a sincere and effective relationship between the rheumatologist and the patient with chronic rheumatic disease is the most efficient and modern method of providing health care services.^11^

The main purpose of this study is to emphasise the need for the development of a medical care model focused on the needs of Greek patients with rheumatic diseases that is based on a relationship of trust and cooperation with their rheumatologist for the best treatment goal.

## RESEARCH INSTRUMENTS

The DoPRAQ-16^12^ (Doctor-Patient Relationship Assessment Questionnaire), assessed the quality of doctor-patient relationship. This questionnaire asks 16 questions to patients and is based on a 5-point Likert scale ranging from 1 (not at all appropriate) to 5 (totally appropriate). From these questions two new scales arise, the scale of positive emotions and the scale of negative emotions, with lower scores in positive emotions translating into higher levels of positive emotions, whereas, on the negative emotion scale lower values indicate higher levels of negative emotions. The scales of positive and negative emotions arise as averages of the answers to the questions, so they take values between 1 to 5 since the individual questions are encoded with values of 1 to 5. There is no cut-off point in either of the two scales; however, the value 3 is a central value in the individual questions. Also, in the questionnaire there is a 17th question in which patients were asked to evaluate their relationship with the doctor on a scale of 1 to 10 where 10 indicates the excellent relationship, 5 that the relationship is moderate and 1 that the relationship is bad.

The Short Form (36) Health Survey is a 36-item patient-reported survey of patient health. The SF-36 consists of eight scaled scores (vitality, physical functioning, physical pain, general health perceptions, body role functioning, emotional role functioning, social role functioning, mental health or emotional wellbeing). Each scale is directly transformed into a 0-100 scale, where the lower score means the more disability and the higher score the less disability without a cut-off point. The eight domains all contribute to physical component summary (PCS) and mental component summary (MCS) scores.^13^

Finally, the Health Assessment Questionnaire (HAQ) was used to assess the functional ability of patients. There were 20 questions in eight categories of functioning which represented a comprehensive set of functional activities – dressing, rising, eating, walking, hygiene, reach, grip, and usual activities. Each category contained at least two specific component questions. The eight category scores were averaged into an overall score on a scale from 0 (no disability) to 3 (completely disabled).^14^

## STATISTICAL ANALYSIS

Statistical analysis was carried out with SPSS v26. The level of statistical significance was set to a=0.05. The one sample Wilcoxon Signed Rank test was used to identify statistically significant differences in the HAQ index with a cut-off value and for both scales of the DoPRAQ-16 questionnaire. Kruskal Wallis-H test was also used for the Physical Functioning, Emotional Role, Social Functioning, and Physical Pain scales of the SF-36 questionnaire to identify statistically significant differences of them in more than two independent groups with post hoc analysis based on non-parametric tests. However, the SF-36 Vitality, Mental Health, Physical Role, and General Health scales do not show extreme outliers or high asymmetry values. Therefore, in order to identify significant differences of them in more than two independent groups One-way ANOVA test with post-hoc analysis using the Tuckey HSD test was used. In addition, bootstrapping correlation with a resampling of 1000 virtual samples was used to test for linear correlation between the DoPRAQ-16 scales and the disability index, as well as between the DoPRAQ-16 and the SF-36 scales. Cronbach’s alpha correlation coefficient was used to assess the reliability of the questionnaires. In terms of:

- DoPRAQ-16 questionnaire, its values ranged between 0.53 (for negative emotions scale) and 0.77 (for positive emotions scale),
- SF-36 questionnaire, its values ranged between 0.81 and 0.92 and
- HAQ questionnaire, its value was 0.94.

All these values varied from acceptable to very good, with the exception of the negative emotion scale, for which the coefficient’s value (alpha=0.53) indicates “poor” reliability. However, this is also an acceptable value.^15^

## DESCRIPTIVE STATISTICAL ANALYSIS

**Figure 1.**
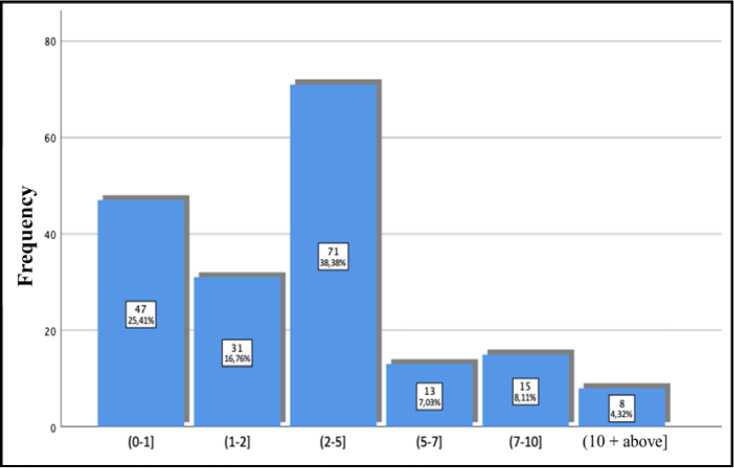
Duration of relationship with the doctor.

The survey was carried out at the General Hospital “KAT” in Athens, Greece. Quantitative research was conducted, using convenience sampling technique. The questionnaire was written in Greek, therefore, the primary criterion for participation in the research was fluency in the Greek language. The survey included 204 patients, suffering from various rheumatic diseases (rheumatoid arthritis, psoriatic arthritis, systemic lupus erythematous e.tc.). Most of them were women (70.1%), married (70.1%), and graduates of compulsory education (36.8%) (**Table 1**). It is also observed that for most of the patients (51 patients, 25%) the duration of the autoimmune disease was (2–5] years, for 36 patients (17.6%) it was (5–10] years and for 39 patients (19.11%) was (10–20] years. Furthermore, as shown in **Figure 1**, the majority of patients (38.4%) had a relationship with their doctor for (2–5) years, followed by (0–1) years (47 patients 25.4%). All participants provided a written consent form as a separate part of the questionnaire, before proceeding with the completion of the survey. Data collection guaranteed anonymity and confidentiality. All subjects were informed of their right to refuse or withdraw from the study, in accordance with the ethical standards of the Committee on Ethics and Research Ethics (UNI.W.A. 102822-17/12/2022).

**Table 1.** Demographic characteristics of survey participants in terms of gender, marital status, education, occupation, and duration of illness.

		**Frequency**	**Percentage (%)**
**Sex**	Man	61	29.9
	Woman	143	70.1
**Marital Status**	Married	143	70.1
	Single	35	17.2
	Divorced / Widowed	26	12.7
**Education Level**	Compulsory Education	75	36.8
	Secondary Education	59	28.9
	University Education	70	34.3
	Science Professions	2	1.0
	Did not Reply	6	2.9
**Duration of illness**	(0–1]	23	11.3
	(1–2]	22	10.8
	(2–5]	51	25.0
	(5–10]	36	17.6
	(10–20]	39	19.1
	(20–30]	13	6.4
	(30-above]	9	4.4
	Did not Reply	11	5.4
**Total**		**204**	**100**

## DESCRIPTIVE IMPRESSION OF THE DIMENSIONS/SCALES OF THE QUESTIONNAIRES

### Questionnaire DoPRAQ-16

Based on **Table 2**, it can be seen that the positive emotions scale has a very low mean value (mean=1.09±0.23), which, according to the guidelines of the questionnaire, indicates a high level of positive emotions regarding the patient’s relationship with his/her doctor. The same table also shows that the scale of negative emotions has a mean value close to five (mean=4.62±0.46), a value that indicates a low level of negative emotions about the patient’s relationship with his/her doctor.

**Table 2.** Descriptive statistics on Relationship Assessment D-P dimensions.

	**Positive Emotions Scale**	**Negative Emotions Scale**
Average	**1.09**	**4.62**
Median	1.00	4.87
Prevailing Rate	1.00	5.00
Standard Deviation	**0.23**	**0.46**
Minimum Rate	1.00	2.88
Maximum Rate	3.13	5.00

### Questionnaire SF-36

**Table 3** shows the descriptive statistics for the scales of the SF-36 Health Survey Questionnaire. Thus, the mean value of Physical Functioning scale is (62.43±27.04) indicating that the patients are not very limited in their physical functions (such as lifting heavy objects, or use the stairs to go up a floor, etc.).

**Table 3.** Descriptive SF-36 dimensional statistics.

	**PF^1^**	**BR^2^**	**ER^3^**	V**^4^**	**MH^5^**	**SF^6^**	**PP^7^**	**GH^8^**
Average	**62.43**	**57.72**	**60.46**	**50.76**	**60.67**	**63.79**	**60.34**	**54.82**
Median	70.00	75.00	66.67	50.00	60.00	62.50	62.00	57.00
Prevailing Rate	75.00	100.00	100.00	45.00	60.00^a^	75.00	62.00	57.00
Standard Deviation	**27.04**	**42.10**	**41.25**	**22.03**	**21.40**	**26.51**	**26.19**	**22.09**
Minimum Rate	0.00	0.00	0.00	0.00	8.00	0.00	0.00	0.00
Maximum Rate	100.00	100.00	100.00	100.00	100.00	100.00	100.00	100.00

Physical Functionality^1^, Body Role^2^, Emotional Role^3^, Vitality^4^, Mental Health^5^, Social Functionality^6^, Physical Pain^7^, General Health^8^

The Physical Role scale has a mean value of (57.72±42.10), which indicates that the patients who participated in the study have mild problems with daily activities that depend on their physical condition, such as doing less than they would like to do, limiting the type of work or activities they do, etc.

Furthermore, the mean value of the Vitality Scale is slightly above 50 (50.76±22.03). This shows that the vitality/energy of the participating patients is not at a good level due to their disease.

The Mental Health of the patients who took part in the survey is relatively good, with a mean value of (60.67±21.40). The patients’ Social Functioning scale also shows a relatively good level as it has a mean value of (63.79±26.51). This suggests that the patients’ social activities were not greatly affected by their health problems, or their psychological state caused by their illness.

With regard to the level of Physical Pain experienced by the patients, the mean value is (60.34±26.19), which indicates that it ranges from moderate to relatively low levels.

Finally, the General Health scale of the questionnaire had a mean value of (54.82±22.09), which is very close to 50. This result indicates that the patients consider their general health to be at a moderate level.

### Health Assessment Questionnaire (HAQ)

The mean disability value for the patients in the study were (0.92±0.43) indicating moderate functioning impairment according to the guidelines. The median value is also quite close to the mean value (median=0.86).

## RESULTS

As mentioned before, regarding the scales/dimensions of the Health-Survey SF-36, there are no cut off points separating abnormal from normal values. However, in the DoPRAQ-16 questionnaire there is a central value (value=3) for individual questions, so that, according to the instructions for the questionnaire, if the questions that make up the positive emotions scale have low values, this indicates a high level of positive emotions, whereas if the questions that make up the negative emotions scale have high values, this indicates a low level of negative emotions.

From the statistical analysis using the non-parametric Wilcoxon signed rank test for one sample, it appears that all the characteristics of the scale of positive emotions such as satisfaction, trust between the doctor and the patient, agreement on the importance of the meetings and the goal of the treatment, have a statistically significant lower value (p<0.05) than the cut-off point (value=3). This suggests that patients have high levels of positive emotions for all these characteristics. The same test also revealed statistically significantly higher value than the cut-off, point (value=3) for all the characteristics of the negative emotions scale. This suggests that the patients had low levels of negative emotions for characteristics such as lack of honesty, discomfort during the meetings and disagreement about the actions.

Bootstrapping correlation with a resampling of 1000 virtual samples was used to assess whether there is a linear correlation between the DoPRAQ-16 scales and the scales of the SF-36 questionnaire. The test showed a weak positive correlation, only between the negative emotions scale and the dimension of physical functioning (Pearson=0.162, p=0.002). This result suggests that a lower level of negative emotions leads to a better level of physical functioning in the patients.

The analysis continued with the non-parametric Kruskal-Wallis H test which was used in order to research statistically significant differences between the categories of duration of the relationship with the doctor and the scales of physical functioning, physical role, emotional role and social functioning. The test was significant (p<0.05) for the dimensions of physical role and social functioning. The following post-hoc analysis with Bonferroni correction showed statistically significant difference (H=−56.65, p=0.043) only for the pair: (2–5], (mean rank=81.16) and (10 and more], (mean rank=137.81) with patients who have had a relationship with their doctor for more than 10 years having a higher average ranking in body role values (**Table 4**).

**Table 4.** Results of post hoc tests, to check differences – homogeneity in the body role per pairs of duration of relationship with the doctor.

**Homogeneous Subsets based on duration of relationship with Doctor**			
1		Subset			
		2	
Sample^a^	(2–5]	81.162	
	(1–2]	90.484	90.484
	(5–7]	92.192	92.192
	(0–1]	101.032	101.032
	(7–10]	105.867	105.867
	(10-above]		137.813
Test Statistic		5.677	6.218
Sig. (2-sided test )		0.225	0.183
Adjusted Sig. (2-sided test)		0.225	0.183

This result suggests that patients whose relationship with the doctor has lasted for more than 10 years have a better quality of health in the dimension of the physical role than patients whose relationship with the doctor has lasted for (2–5] years.

A similar analysis was carried out for the social functioning scale, in order to identify those pairs of relationship durations with the doctor that show significant differences, the results of which are presented in **Table 5**.

**Table 5.** Results of post hoc tests to check for differences – homogeneity in social functioning per pairs of relationship duration with the doctor.

**Homogeneous Subsets based on duration of relationship with docto**r			
1		Subset	
		2	
Sample	(5–7]	77.038	
	(2–5]	83.380	
	(0–1]	89.106	89.106
	(7–10]	102.800	102.800
	(1–2]	114.290	114.290
	(10-above]		126.313
Test Statistic		9.008	5.600
Sig. (2-sided test )		0.061	0.133
Adjusted Sig. (2-sided test)		0.061	0.192

In **Table 5** it is clear that the pairs: a) (5–7], (Mean Rank=77.04) and (10 and above], (Mean Rank=126.31) and b) (2–5], (Mean Rank=83.38) and (10 and above], (Mean Rank=126.31), have statistically significant differences*,* with patients who have been in a relationship with their doctor for more than 10 years have a better quality of life in terms of social functioning than patients who have been in relationship with their doctor for (2–5] and (5–7] years.

As regards the duration of the relationship with the doctor, the analysis continued with the parametric One-Way ANOVA test which was statistically significant for the dimensions of vitality df(5, 179)=2.622, p=0.026 and general health df(5, 179)=2.639, p=0.041. Therefore, a post-hoc analysis using the Tukey test was carried out in order to identify those pairs of relationship durations with the doctor that showed significant differences in these dimensions. The results of the analysis showed that statistically significant differences exist:

- for the vitality dimension (p=0.02), only for the pair (2–5] (M=47.32, SD=20.97) and (10 and above], (M=73.13, SD=16.89) and
- for the general health dimension (p=0.04), only for the pair (2–5] (M=50.39, SD=22.14) and (10 and above], (M=74.88, SD=17.50).

Patients with a relationship with their doctor of more than 10 years have a better quality of health for both dimensions, than patients with a relationship of (2–5] years.

## DISCUSSION

In recent years, interesting developments have taken place in the field of rheumatology. There are important new insights into the pathogenesis, diagnosis, and prognosis of autoimmune diseases. New innovative treatments are used in clinical practice and have remarkably improved the lives of rheumatic patients. The “journey” of a patient with rheumatological disease from the appearance of the first symptoms to the final diagnosis and treatment has become quite short. An important role in this seems to be played by the increasing public awareness of the symptoms of rheumatic diseases, by medical companies and patient associations, which try to direct the rheumatic patient to a rheumatologist as early as possible. Rheumatic diseases are chronic systemic inflammatory diseases which, if left untreated through the mechanism of inflammation, they lead to the destruction of the joints, resulting in the loss of physical functionality and the inability to perform daily activities. The therapeutic approach has changed radically in recent years. The explosion in the field of biotechnology has made it possible through research to better understand the pathogenetic pathways of autoimmune diseases and to develop targeted therapies. Rheumatologists now have biological agents in their therapeutic arsenal, which significantly slow down the progression of the disease. The use of these drugs ensures patients a life without pain, fewer side effects and better functionality.^16^

The world’s major organisations publish guidelines for the treatment of rheumatic diseases (ACR/EULAR), aiming at clinic-laboratory remission or low disease activity. The achievement of clinical remission, which is achieved with the new therapeutic means, slows down their radiological progression, prevents the appearance of permanent joint deformities, minimises symptoms and especially pain and generally improves the quality of life of patients. In the past, we simply monitored the progression of rheumatic diseases, minimising only the pain. Now, we are able to intervene substantially, resulting in an optimisation of the patient’s situation.^17^

However, while intensive scientific research has led to advances in the diagnostic and therapeutic approach to autoimmune diseases, there are unmet needs in the area of communication between the doctor and the patient as a key parameter for the treatment of the disease. In rheumatic patients, these diseases, apart from the physical symptoms, also cause an increased psychological burden, so that a meaningful communication and a harmonious cooperation are of great importance for a more accurate evaluation of the disease and the most appropriate therapeutic approach. Cooley^18^ gave the first definition of the term communication as “the mechanism through which human relationships exist and develop” and which is inextricably linked to all human activity. The rheumatologist, after informing the patients in detail in a simple and understandable way, should give the space and time to accept their chronic problem and then, with their active participation, they should treat it together based on the treatment protocols of each disease, but also individualising the options for each patient. The doctor should be a companion with the patient in this difficult and chronic journey, based on honesty, trust and collaboration.

According to Papadakis and Michailidis,^19^ communication takes place through the verbal and non-verbal pathway, of which the verbal pathway expresses the cognitive part of communication and the non-verbal the emotional one. The international literature and the scientific community recognise that the main qualifications for information gathering, diagnosis, and treatment are the communication skills that the doctor must possess, namely active listening and interpretation of non-verbal messages.

Several studies have shown that physicians who are aware of non-verbal communication methods have the ability to gain a high degree of patient satisfaction and cooperation in following instructions.

The literature emphasises the negative impact of rheumatic diseases on the physical, psychological, and social functioning of patients. When the symptoms occur in the hands of patients, daily activities such as writing, opening jars, dressing, and carrying objects are affected, while when the feet are affected, patients find it difficult to walk and, in general, to move around. Patients are also particularly concerned about how chronic rheumatic disease will affect their lives, and the risk of losing their independent living. This insecurity also has a serious impact on their mental health. Studies on the doctor-patient relationship^20–22^ showed that better communication is associated with better health, less organ damage, lower disease activity and fewer drug side effects. In this study, the above findings were verified through quantitative research, concluding that lower levels of negative emotions lead to better levels of physical functioning in patients with rheumatic diseases.

It is the doctor who will assess the course of the patient’s disease and give him or her the appropriate treatment. The development of a relationship that aims to create a bond between the two parties and involves the definition of therapeutic objectives and the completion of therapeutic activities is defined as a therapeutic alliance. Therapeutic communication focuses on the needs of the patient. The person-centred approach establishes a good relationship with the patient that facilitates the exchange of information, understanding of this information and regulation of the patient’s emotions. Kneisel^23^ emphasised that “this relationship implies the involvement of both the specialist and the patient, focusing on the patient and his/her living conditions, with the aim of improving them”. The literature shows that when patients’ satisfaction with their relationship with their doctor is achieved, compliance with medication is ensured. When patients feel comfortable and trust the rheumatologist, they have better levels of health, whereas poorer health is associated with lower levels of trust.^24^

One factor explored in this thesis to play an important role in the patient’s health status is the length of relationship with the doctor. A recent study^25^ found that trust in the doctor develops over time and characterises the long-term doctor-patient relationship. In the present quantitative study, a positive correlation was observed between the length of the doctor-patient relationship and health quality. Specifically, patients whose duration of relationship with the doctor more than 10 years, have better health quality in the dimension of Physical Role and Social Functioning compared to people whose relationship with the doctor lasts 2–5 years. An integrated relationship with the doctor is slowly built up over the years and should be the cornerstone of daily medical practice. It is not enough for a doctor to be scientifically competent, but it is also essential that his or her humane side is brought out through understanding, respect, and support for the patient. The doctor is no longer the authority who decides on the patient’s treatment. In recent years, the doctor and the patient have played an equal role in the choice of treatment. The therapeutic alliance is an element of the therapeutic relationship that involves both the development of therapeutic boundaries (respect, trust, appreciation) and the establishment of therapeutic goals. Together both will co-decide on the therapeutic approach to fight the common enemy, which is the disease.
